# Diminution of the gut resistome after a gut microbiota-targeted dietary intervention in obese children

**DOI:** 10.1038/srep24030

**Published:** 2016-04-05

**Authors:** Guojun Wu, Chenhong Zhang, Jing Wang, Feng Zhang, Ruirui Wang, Jian Shen, Linghua Wang, Xiaoyan Pang, Xiaojun Zhang, Liping Zhao, Menghui Zhang

**Affiliations:** 1 State Key Laboratory of Microbial Metabolism, Joint International Research Laboratory of Metabolic & Developmental Sciences, and School of Life Sciences and Biotechnology, Shanghai Jiao Tong University, Shanghai, 200240, P.R. China

## Abstract

The gut microbiome represents an important reservoir of antibiotic resistance genes (ARGs). Effective methods are urgently needed for managing the gut resistome to fight against the antibiotic resistance threat. In this study, we show that a gut microbiota-targeted dietary intervention, which shifts the dominant fermentation of gut bacteria from protein to carbohydrate, significantly diminished the gut resistome and alleviated metabolic syndrome in obese children. Of the non-redundant metagenomic gene catalog of ~2 × 10^6^ microbial genes, 399 ARGs were identified in 131 gene types and conferred resistance to 47 antibiotics. Both the richness and diversity of the gut resistome were significantly reduced after the intervention. A total of 201 of the 399 ARGs were carried in 120 co-abundance gene groups (CAGs) directly binned from the gene catalog across both pre-and post-intervention samples. The intervention significantly reduced several CAGs in *Klebsiella*, *Enterobacter* and *Escherichia*, which were the major hubs for multiple resistance gene types. Thus, dietary intervention may become a potentially effective method for diminishing the gut resistome.

Since the 1940s, the use of antibiotics has greatly reduced infectious mortality and increased life expectancy. However, antibiotic resistance has developed rapidly in microbes and has become one of the greatest global threats to human health, leading to increasing healthcare costs, prolonged hospital stays, treatment failure and death^1^.

Many previous studies on antibiotic resistance have focused on clinical pathogens, but recent efforts have shifted to the “resistome”, which refers to the collection of all the antibiotic resistance genes (ARGs) in a particular environment^2^. The human gut is a reservoir of ARGs^3^, which can be exchanged among the members of the microbiota including both commensals and opportunistic pathogens^4^. Advances in DNA sequencing have enabled us to explore the human gut resistome at the whole microbial community level. Large adult cohort studies using metagenomics to examine the human gut resistome have identified thousands of ARGs in gut bacteria^5^.

Dietary intervention is a common measure used to reduce body weight in obese individuals. Diet is also known as a major force that shapes the gut microbiota^6^,^7^. However, how dietary interventions intended for body weight control influence the gut resistome remains elusive. In our previous dietary interventional study (See Methods part for details), we showed that a diet composed of whole grains, traditional Chinese medicinal foods and prebiotics (WTP diet) significantly reduced body weights and improved clinical parameters and inflammatory conditions in children with both genetic and simple obesity. The intervention also significantly modified the composition and function of the gut microbiota, including a decrease in toxin producers and an increase in beneficial bacteria^8^. In this study, using a genome-centric metagenomic approach, we systemically compared the gut resistome of 35 obese children before and after intervention and found that dietary intervention significantly diminished the gut resistome of these individuals.

## Results

### The altered profile of antibiotic resistance genes (ARGs)

Three hundred and ninety nine ARGs were identified from the gene catalog of ~2 million human gut microbial genes, which was established in a previous study^8^ using a BLAST search against the core set of antibiotic resistance protein sequences from the Antibiotic Resistance Genes database (ARDB)^9^, under a determined threshold^5^.

**Figure 1:**
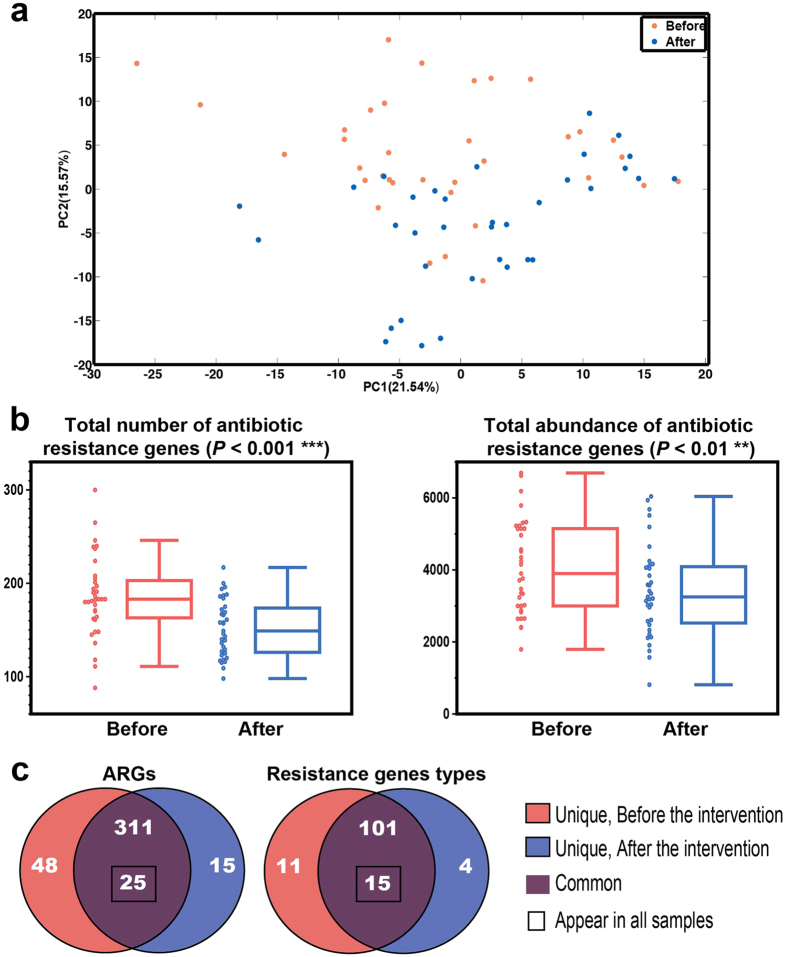
The overall structural alterations of the gut resistome after intervention. **(a)** The PCA score plot based on the profile of 399 ARGs showing significant segregation between the samples before and after dietary intervention (log-transformed, PERMONOVA P = 0.0187, permutations = 9999). **(b)** The total number and abundance of ARGs. The boxes denote the interquartile range (IQR) between the first and third quartiles (25th and 75th percentiles, respectively), and the line inside the boxes denotes the median. The whiskers denote the lowest and highest values within 1.5 times of the IQR from the first and third quartiles, respectively. The samples are displayed as the dot on the left. Statistical analysis was performed with a two-tailed Wilcoxon matched-pairs signed rank test (n = 35). **(c)** The prevalence of ARGs and ARG responding to resistant gene types.

The Principal Component Analysis (PCA) score plot of the ARG profile showed a significant segregation between samples taken before and after the intervention (Fig. 1a) (permutational MANOVA test, 9999 permutations, *P* = 0.0187). The total number of ARGs decreased from 185.54 ± 10.56 (the data are expressed as the mean ± s.e.m. unless otherwise stated) to 151.91 ± 7.37 (Fig. 1b). In the post-intervention samples, 48 ARGs disappeared while 15 new ARGs appeared (Fig. 1c), which suggests dynamic gains and losses of ARGs during the intervention. Although new ARGs appeared, the number of samples (1.6 ± 1.1, mean ± s.d.) carrying them was lower than the number of samples carrying those that disappeared (2.9 ± 2.1, mean ± s.d.).

**Figure 2:**
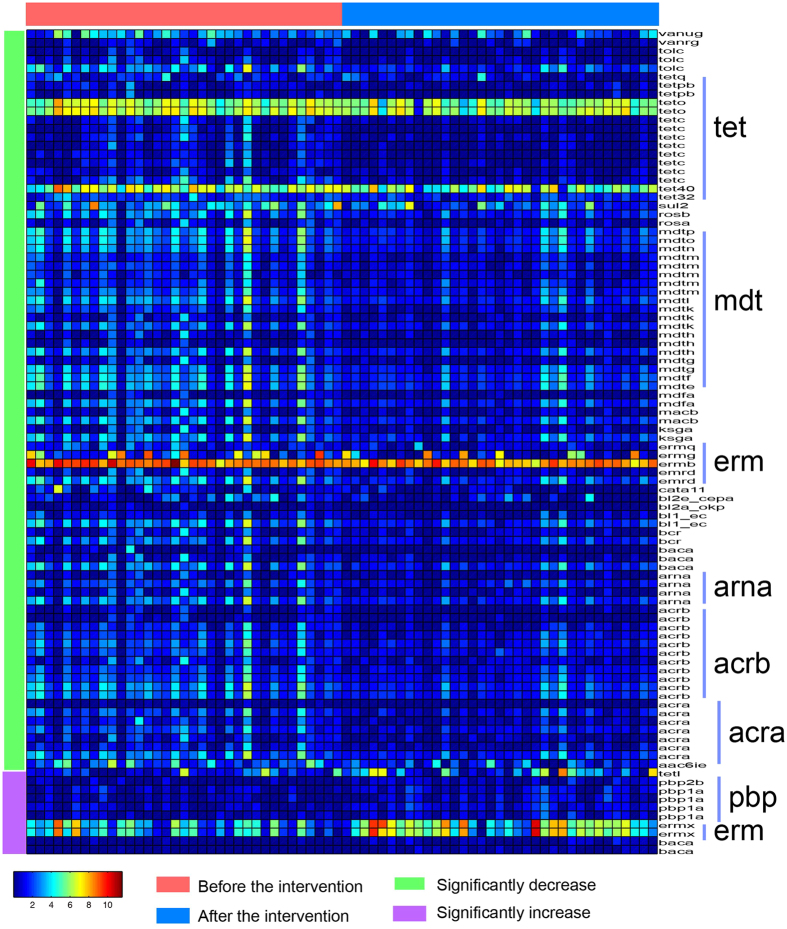
A heatmap of 96 significantly changed ARGs and their responses to the dietary intervention. The color of the spot corresponds to the log-transformed abundance of the ARGs. The ARGs are organized according to antibiotic resistant gene types.

Furthermore, the total abundance also significantly decreased after the dietary intervention from 4075.51 ± 313.91 to 3410.52 ± 306.90 (Fig. 1b). Among the 399 ARGs, the abundance of 86 ARGs significantly decreased and accounted for 33.39% ± 3.39% and 24.48% ± 2.79% of the total resistome abundance in each individual before and after the intervention. A total of 222 ARGs decreased and accounted for 47.90% ± 4.48% (before) and 41.22% ± 5.68% (after) of the resistome. Although 10 ARGs increased significantly, their abundance only increased from 1.96% ± 0.94% (before) to 8.94% ± 3.33% (after). The ARGs that significantly decreased mainly belonged to the tet, mdt, and erm families, and the majority of the increased ARGs were members of the pbp and erm families (Fig. 2 and Supplementary Table 1).

### The altered resistance gene types and corresponding antibiotics

**Figure 3:**
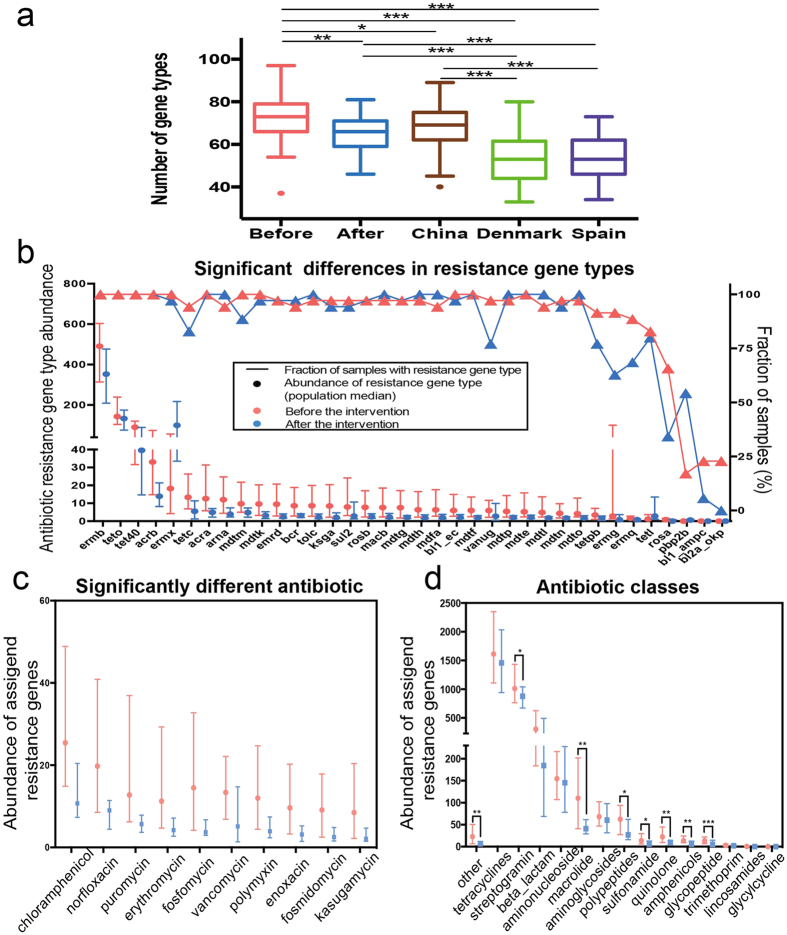
The antibiotic resistance genes that significantly changed after intervention. (**a**) The number of antibiotic resistance gene types in our intervention trial (before and after) and three other cohorts (China, Denmark and Spain from ref. 5). The boxes denote the interquartile range (IQR) between the first and third quartiles (25th and 75th percentiles, respectively), and the line inside the boxes denotes the median. The whiskers denote the lowest and highest values within 1.5 times of the IQR from the first and third quartiles, respectively. (**b**) The 36 significantly changed gene types. The lines/triangle markers represent the fractions of samples before and after the intervention. The dot/bar markers represent the median and 25%/75% quartiles for the abundance of the gene types. (**c**) The significantly changed abundance (adjusted *P* < 0.05) of ARGs assigned to antibiotic compounds. Resistance genes were assigned to antibiotics according to the ARDB. The abundances of genes conferring resistance for the same antibiotic compound were summed. (**d**) The abundance of ARGs assigned to each antibiotic class. Resistance genes were assigned to antibiotic classes according to the ARDB. The abundances of the genes conferring resistance for the same antibiotic class were summed. Statistical analysis for (**a**) pairwise comparisons was performed with a two-tailed Mann–Whitney U test and a two-tailed Wilcoxon matched-pairs signed rank test (before n = 35, after n = 35, China n = 38, Denmark n = 85, and Spain n = 39); for (**b**–**d**), a Wilcoxon matched-pairs signed rank test (n = 35) was performed. *Adjusted P < 0.05, **Adjusted P < 0.01, ***Adjusted P < 0.001 (Benjamini & Hochberg 1995).

The 399 ARGs were grouped into 131 different resistance gene types, of which 15 gene types were found in all samples including TetW, TetQ, TetO, TetM, Tet40, Tet32, MdtF, MdtE, ErmF, ErmB, Bl2e_cfxa, BacA, AcrB, AcrA and Aac6Ie. The 11 and 4 gene types were unique in the pre- and post-intervention samples, respectively (Fig. 1c). The number of gene types in the pre-intervention samples was significantly higher than that in adults from China, Denmark and Spain (Fig. 3a). After the intervention, the number was significantly reduced and showed no significant difference compared with that of Chinese adults but was still higher than the number of gene types for Danish and Spanish adults (Fig. 3a).

Out of the 131 gene types, the abundances of 36 were significantly changed after the intervention (Fig. 3b). On average, for the significantly decreased 33 gene types, their summed abundance was reduced from 1364.2 ± 188.85 to 761.6 ± 78.81 for each individual. Only three gene types significantly increased, including ErmX for lincosomide, macrolide and streptogramin b, TetL for tetracycline and PBP2b for penicillin. A total of 8 of these 36 gene types remained unchanged and 16 were reduced in prevalence (Fig. 3b).

When all of the gene types were mapped to their corresponding antibiotic compounds, we obtained the potential resistance profiles for 47 antibiotics. A total of 10 out of the 47, namely chloramphenicol, norfloxacin, puromycin, erythromycin, fosfomycin, vancomycin, polymycin, enoxacin, fosmidomycin and kasugamycin, were significantly changed and all of them were reduced after the intervention (Fig. 3c). At the antibiotic class level, the abundances of 8 out of the 15, including streptogramin, macrolide, polypeptides, sulfonamide, quinolone, amphenicols and glycopeptide, were significantly reduced in the post-intervention samples (Fig. 3d).

The ARGs of bacteria mainly perform their functions through three resistance mechanisms: 1) reducing the intracellular concentrations of antibiotics via poor penetration or efflux, 2) modifying the antibiotic target for protection, and 3) hydrolyzing or modifying the antibiotic for inactivation^10^. According to the ARDB, the ARGs were assigned to the three resistance mechanisms. After the intervention, the abundances of target protection and the efflux pumps significantly decreased (Supplementary Fig. S1). Among the erythromycin ribosome methylase (erm) family, which prevents the binding of macrolides, lincosamines and streptogramins by methylating 16S rRNA^11^, dietary intervention significantly reduced the abundances of ErmB, ErmG and ErmQ. Moreover, the abundances of TetO and TetPB, which protect the ribosome from the translation inhibition of tetracycline, decreased significantly after the intervention. Other significantly reduced gene types that conferred target protection included sul2, arna, ksga and vanug. Bacterial efflux pumps can transport many antibiotics out of the cell, thus reducing the intracellular concentrations of antibiotics. After the intervention, the abundances of ErmD and 10 Mdt (E, F, G, H, K, L, M, N, O, P) multidrug resistance efflux pumps and the AcrAB-TolC multidrug efflux complex^12^ decreased significantly, which suggests the reduction of potentially multidrug resistant bacteria. Furthermore, specific efflux pumps, including Bcr for bacitracin, MacB for macrolide, Tet40 and TetC for tetracycline and the RosA-RosB complex for fosfomycin, also significantly decreased after the intervention.

### Identification of ARG carriers

In our previous study, the gene catalog was binned into co-abundance gene (CAG) groups using a canopy-based algorithm on the basis of the correlation with gene abundance^8^,^13^. CAGs were further classed into two categories according to the number of genes within them. Large CAGs >700 genes are considered bacterial genomes and small CAGs ≤700 genes may potentially exist as incomplete bacterial genomes or small genetic entities such as phages^13^. Within the resistome, 201 ARGs, whose average accounted for 86.60% ± 1.11% and 87.45% ± 1.99% of total resistome abundance in each individual before and after the intervention, originated from 38 large CAGs and 80 small CAGs. On average, the total abundance of ARGs in large CAGs was 584.88 ± 129.47 (before) and 334.54 ± 61.70 (after) and 2981.58 ± 246.93 (before) and 2665.39 ± 272.79 (after) in small CAGs. A total of 198 ARGs were not assigned to CAGs. The total abundance of these ARGs significantly decreased from 532.63 ± 60.82 to 425.13 ± 103.35 after the intervention.

We performed de novo assembly for each large CAG that carried ARGs (see the methods). A total of 24 of the genome assemblies met at least five of the six quality criteria for draft genomes determined by the Human Microbiome Project (HMP). Using CVtree3.0 web server^14^ and specI^15^, the phylogenies of these draft genomes were identified^8^. The taxonomic assignments of the remaining large CAGs and 80 small CAGs were performed via comparison to the reference genomes (see methods), for which 8 large CAGs and 22 small CAGs were assigned to the species/genus level. Among the 118 ARG-carrying CAGs, the top three were CAG00002 (*Escherichia/Shigella sp.*), CAG00001 and CAG00008 (*Klebsiella pneumonia*), which carried 41, 37 and 20 ARGs, respectively. As a result of the comparison to the reference genomes at the nucleotide level, 51.5% of the genes of CAG00001 were assigned to *Escherichia/Shigella*, 16.7% to *Weissella* and 14.9% to *Enterococcus*, which indicated that CAG00001 was still heterogeneous. Thus, we performed gene binning of CAG00001 one more time to separate the genomes by increasing the parameter “–max_canopy_dist” in the Canopy tool from 0.9–0.95. Four subgroups labeled CAG00001a ~ d were obtained. CAG00001a and c were assigned to *Escherichia/Shigella sp*., whereas CAG00001b and d could not be assigned at the species/genus level. CAG00001a, b, c and d carried 28, 3, 5 and 0 ARGs, respectively. Three ARGs were not assigned to CAG00001a ~ d due to the stricter binning parameters. As one gene can belong to multi-CAGs, the total number of ARGs in CAG00001a ~ d is larger than 34.

Thus, on the basis of gene binning, assembly and taxonomy assignment, we eventually identified 120 (CAG00001a ~ c + the rest of 117 ARG carrying CAGs) ARG carriers. The PCA score plot of the ARG carrier profile showed a trend for separation between the before and after intervention samples (Supplementary Fig. S2) (permutational MANOVA test, 9999 permutations, *P* = 0.0584). The procrustes analysis combining the PCA of the ARG carriers with that of the 399 ARGs showed that the shift in these carriers was significantly associated with the altered resistome (Monte-Carlo test, *P* < 0.001, M^2^ = 0.23). Considering the high abundance of ARGs that the 120 carriers harbored, this result suggests that they mainly contributed to the altered resistome structure.

### The distribution network of ARGs and their carriers

**Figure 4:**
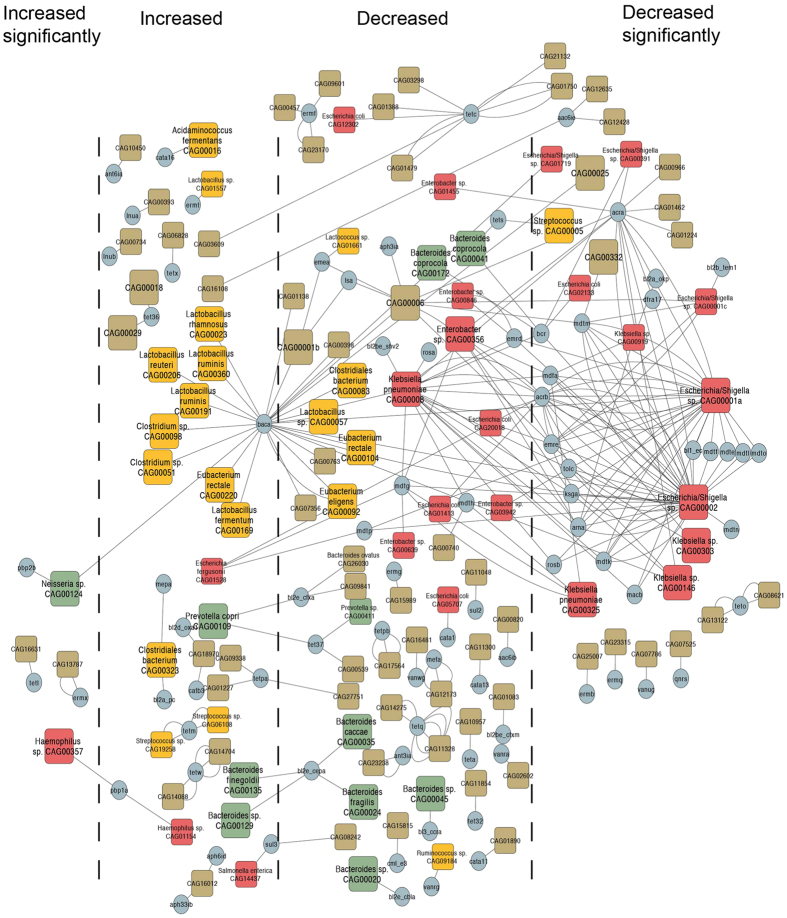
The distribution network of ARGs in the ARG carriers. The colors of the squares represent different phyla (Red: Proteobacteria, Green: Bacteroidetes, and Yellow: Firmicutes). The sizes of the squares represent the two categories of ARG carriers (Large: >700 genes and Small: ≤700 genes). The direction of the change in abundance for each ARG carrier was separated into four categories, of which the significance was determined by the adjusted P < 0.05 (a two-tailed Wilcoxon matched-pairs signed rank test, n = 120, Benjamini & Hochberg 1995).

Based on the direct attribution between ARGs and their carriers, the distribution network of ARGs in this particular cohort was constructed (Fig. 4). The ARGs existed across multiple phyla including Firmicutes, Bacteroidetes and Proteobacteria, which indicated a wide distribution of ARGs in the bacterial kingdom. Bacteria in Firmicutes carried the minimum load of ARGs, and each bacterium mostly carried only one gene type. The gene type BacA conferring resistance to bacitracin was carried extensively in Firmicutes and was an obvious hub in the network. A total of 6 *Lactobacillus*, 3 *Eubacterium* and 3 Clostridiales bacteria carried this gene type. The bacteria in Bacteroidetes mainly (8/11) carried gene types conferring resistance to beta-lactamase antibiotics including php2b, BL3_ccra, BL2e_cbla, BL2e_cepa and BL2e_cfxa. The gene type BL2e_cepa only existed in the genus *Bacteroides*, which was consistent with the content in ARDB, which suggested the potential taxonomic specificity of this gene type. The members of Enterobacteriaceae bacteria, especially the *Klebsiella*, *Enterobacter* and *Escherichia* genera, were the hubs in the network. The 10 mdt (E, F, G, H, K, L, M, N, O, and P) multidrug resistance efflux pumps and the AcrAB-TolC multidrug efflux complex were carried in these genera specifically, which indicated the multidrug resistance of these bacteria. Moreover, these bacteria carried gene types conferring resistance to a wide range of antibiotics such as beta-lactam, tetracycline, sulfonamide, and macrolide. In addition, after intervention, the abundance of these bacteria, especially CAG00001a (*Escherichia/Shigella sp.*), CAG00002 (*Escherichia/Shigella sp*.), CAG00146 (*Klebsiella sp.*), CAG00008 (*Klebsiella pneumoniae*) and CAG00356 (*Enterobacter sp*.), decreased significantly or followed a similar trend.

## Discussion

A previous study showed that Chinese adults harbored more diverse and abundant ARGs than Danish and Spanish adults^5^. Here, we found a gut resistome with significantly more ARG types in obese children before intervention than Chinese adults. Moreover, the prevalence of TetW, TetQ, TetO, TetM, Tet40, Tet32, ErmB and BacA in all samples was consistent with previous studies in adults^5^,^16^,^17^, which indicated the wide distribution of tetracycline, erythromycin and bacitracin resistance genes among obese children. As in the adult gut resistome, tetracycline resistance genes were the most abundant in the obese children before intervention^5^. In the fecal samples of healthy infants and children, diverse and novel ARGs have been identified^18^. A recent large cohort study measured the total urinary concentration of 18 antibiotics from 1,064 Chinese school students and showed that these children carry a heavy antibiotic burden^19^, which is closely related to the extensive exposure to antibiotics in Chinese children. Obese children might have additional problem, since obese individuals are at a higher risk for infection^20^, receive more frequent antibacterial treatment^21^ and have more antibiotic treatment failure^22^. Thus, it is not surprising to find that the obese children in our study harbor a more diverse gut resistome than Chinese adults.

Gene-centric metagenomic analysis is the general approach that has been applied to probe the gut resistome in previous studies^5^,^23^,^24^. Using this method with deep metagenomic sequencing, we studied the resistome of obese children before and after the intervention at different levels including ARGs, gene types and resistance potential to corresponding antibiotics and antibiotic classes. Our results showed that dietary intervention eliminated 48 ARGs and reduced the abundance of more than 77% ARGs significantly or following a trend. These reduced ARGs conferred resistance to a wide range of antibiotics. Thus, after the intervention, both the richness and diversity of the gut resistome were significantly diminished. We also notice the larger variability in abundance of ARGs in the pre-treatment samples. This wide variation could perhaps be mined with respect to what lifestyle, medical, genetic or other factors are associated with the ‘resistome’ load. Although we cannot fully draw conclusions about what is driving the variability, we find that the dietary intervention can narrow it.

The ARGs belonging to the same gene types, such as those in the erm family, demonstrated different changing directions, which could be a result of the different responses of their carriers to dietary intervention. Thus, it is important to assign ARGs to their corresponding carriers, which are both hosts for executing resistance functions and targets for dietary intervention. A genome-centric metagenomic analysis can provide direct insight into the function and physiology of individual community members by reconstructing bacterial genomes in environmental samples^25^. With the Canopy-based algorithm, we assigned 201 ARGs to 120 co-abundance gene groups. These groups were of interest because they were potential carriers of antibiotic resistance genes. By sequential de novo assembly and taxonomic annotation, we obtained the draft genomes and their phylogenies.

We constructed an ARG distribution network for their carriers. This network directly provided the types and numbers of ARGs in each bacterium in our dataset without the need to search for attribution information in reference databases. In the distribution network, the heavy load of ARGs in the *Klebsiella*, *Enterobacter* and *Escherichia* taxa was observed and these ARG carriers existed as hubs connecting multiple resistance gene types. This observation was consistent with previous studies highlighting the severe antibiotic resistance of these bacteria^26^,^27^, which indicated that multidrug resistant opportunistic pathogens also existed in our studied cohort. As reported, infections caused by multidrug-resistant pathogens lead to not only higher mortality and more treatment failures but are also more costly for health systems^28^. In this study, we found that dietary intervention significantly (or at least following a trend) reduced 69% of ARG carriers, especially those with multidrug resistance.

The WTP diet used in the intervention is rich in non-digestible carbohydrate. With respect to the unrestricted diets before the intervention, it narrows the nutrition resources and shifts the fermentation of bacteria from protein to carbohydrate in the gut^8^. As the results, the bacteria with high carbohydrate utilizing ability were enriched such as *Bifidobacterium*^8^ and therefore affect other bacteria through potential interactions or extracellular metabolites. Our study finds that most of the increased ones have few or no ARGs and the decreased ones harbored heavy load of ARGS, which leads to the overall diminution of the gut resistome.

Many efforts have been made to manage the threat of antibiotic resistance from different perspectives. Our results indicate that gut microbiota-targeted intervention not only significantly modified the gut microbiota of obese children and improved their health but also significantly diminished the resistome. The reduction of the resistome abundance together with the decreased number of the ARG members and particularly the diminution of multiple resistance ARG carriers suggests that dietary intervention could be a potential solution for this problem.

## Materials and Methods

### Clinical Investigation

Our previous study was performed under the approval of the Ethics Committee of the School of Life Sciences and Biotechnology, Shanghai Jiao Tong University (No. 2012–016) and carried out in accordance with the approved guidelines. The clinical trial was registered at Chinese Clinical Trial Registry (No. ChiCTR-ONC-12002646). Written informed consents were obtained from the guardians of all the participant children.

### Datasets

In our previous study, children with genetic (*n* = 17) and simple (*n* = 21) obesity received gut microbiota-targeted dietary intervention with the diet composed of whole grains, traditional Chinese medicinal foods and prebiotics. Illumina-based metagenomic sequencing was performed on the fecal samples collected from these individuals (Day 0 and 30 for simple obesity; Day 0, 30, 60 and 90 for genetic obesity)^8^. The original data can be downloaded from the NCBI SRA database (accession number: SRP045211). In this matched-pair study, we excluded three individuals due to incomplete data (individual GD10 without data on Day 30) or because they were diagnosed as outliers (individuals GD20 and GD26). The metagenomic data set had an average of 84.6 million ± 21.2 million (mean ± s.d.) high quality reads, which was based on the remaining 35 obese children on Day 0 and Day 30.

### Identification of antibiotic resistance genes (ARGs)

The core set of 7,828 antibiotic resistance proteins from the Antibiotic Resistance Database (ARDB) was downloaded (ftp://ftp.cbcb.umd.edu/pub/data/ARDB/ARDBflatFiles.tar.gz). All of the proteins from our non-redundant gene catalog, which was previously constructed^8^, were aligned with these antibiotic resistance proteins using BLASTP with an E-value threshold of 1e-10, query coverage of at least 70% and an identity of at least 80%^5^.

### The abundance of ARGs

The abundance of the ARGs was the subset of the abundance profile of the gene catalogue, which was calculated in our previous study^8^. In brief, the abundance was calculated from the alignment of high quality reads, corrected by sequencing depth and calculated as gene-length normalized base counts.

### The assembly of large (co-abundance gene groups) CAGs

All of the genes, of which the total abundance was distributed in more than 3 samples, were clustered into CAGs based on their abundance using a Canopy-based algorithm^13^. All of the large CAGs, which carried ARGs, were assembled as previously described^8^. In brief, the sample-specific and CAG-specific read set was obtained by aligning reads to the CAG-specific contigs and then de novo assembled with Velvet. To assess the quality of the assemblies, we adopted the six high-quality draft genome assembly criteria from the Human Microbiome Project (HMP) (http://www.hmpdacc.org/reference_genomes/finishing.php).

### Taxonomic assignment of CAGs

For the large CAGs with high quality draft genomes meeting at least 5 HMP criteria, we used the CVtree3.0 web server and SpecI to determine the phylogeny^8^. The taxonomic assignments of small CAGs and large CAGs with low quality draft genomes were performed by aligning the genes in each CAG to 7,991 reference genomes from the NCBI database (downloaded on May 6, 2014) at both the nucleotide (BLASTN) and protein (BLASTP) levels. The alignments were filtered by the E-value (<1e-10 for the nucleotide level and <1e-5 for the protein level) and query coverage (>70%). Following the taxonomic assignment threshold described previously^29^, the CAGs were assigned to the species or genus level (species level: 90% of genes can be mapped to the species’ genome with >95% identity at the DNA level; genus level: 80% of genes can be mapped to a genus with >85% identity at both the DNA and protein levels).

## Additional Information

**How to cite this article**: Wu, G. *et al.* Diminution of the gut resistome after a gut microbiota-targeted dietary intervention in obese children. *Sci. Rep.* **6**, 24030; doi: 10.1038/srep24030 (2016).

## Supplementary Material

Supplementary Information

## References

[b1] World, Health & Organization. *Antimicrobial Resistance*: *Global Report on Surveillance* (2014).

[b2] D’Costa V. M., McGrann K. M., Hughes D. W. & Wright G. D. Sampling the antibiotic resistome. Science 311, 374–377, 10.1126/science.1120800 (2006). 16424339

[b3] Sommer M. O. A., Dantas G. & Church G. M. Functional Characterization of the Antibiotic Resistance Reservoir in the Human Microflora. Science 325, 1128–1131, 10.1126/Science.1176950 (2009). 19713526 PMC4720503

[b4] Stecher B. *et al.* Gut inflammation can boost horizontal gene transfer between pathogenic and commensal Enterobacteriaceae. P Natl Acad Sci USA 109, 1269–1274, 10.1073/Pnas.1113246109 (2012). PMC3268327 22232693

[b5] Hu Y. F. *et al.* Metagenome-wide analysis of antibiotic resistance genes in a large cohort of human gut microbiota. Nat Commun 4, Artn 2151, 10.1038/Ncomms3151 (2013). 23877117

[b6] Zhao L. The gut microbiota and obesity: from correlation to causality. Nat Rev Microbiol 11, 639–647, 10.1038/nrmicro3089 (2013). 23912213

[b7] Xu Z. J. & Knight R. Dietary effects on human gut microbiome diversity. Brit J Nutr 113, S1–S5, 10.1017/S0007114514004127 (2015). 25498959 PMC4405705

[b8] Zhang C. *et al.* Dietary modulation of gut microbiota contributes to alleviation of both genetic and simple obesity in children. EBioMedicine 2, 966–982, 10.1016/j.ebiom.2015.07.007 (2015). 26425705 PMC4563136

[b9] Liu B. & Pop M. ARDB-Antibiotic Resistance Genes Database. Nucleic Acids Res 37, D443–D447, 10.1093/Nar/Gkn656 (2009). 18832362 PMC2686595

[b10] Blair J. M. A., Webber M. A., Baylay A. J., Ogbolu D. O. & Piddock L. J. V. Molecular mechanisms of antibiotic resistance. Nat Rev Microbiol 13, 42–51, 10.1038/Nrmicro3380 (2015). 25435309

[b11] Leclercq R. Mechanisms of resistance to macrolides and lincosamides: Nature of the resistance elements and their clinical implications. Clin Infect Dis 34, 482–492, 10.1086/324626 (2002). 11797175

[b12] Perez A. *et al.* Cloning, nucleotide sequencing, and analysis of the AcrAB-TolC efflux pump of Enterobacter cloacae and determination of its involvement in antibiotic resistance in a clinical isolate. Antimicrobial agents and chemotherapy 51, 3247–3253, 10.1128/AAC.00072-07 (2007). 17638702 PMC2043211

[b13] Nielsen H. B. *et al.* Identification and assembly of genomes and genetic elements in complex metagenomic samples without using reference genomes. Nat Biotechnol 32, 822–828, 10.1038/Nbt.2939 (2014). 24997787

[b14] Qi J., Wang B. & Hao B. I. Whole proteome prokaryote phylogeny without sequence alignment: A K-string composition approach. J Mol Evol 58, 1–11, 10.1007/S00239-003-2493-7 (2004). 14743310

[b15] Mende D. R., Sunagawa S., Zeller G. & Bork P. Accurate and universal delineation of prokaryotic species. Nat Methods 10, 881−+, 10.1038/Nmeth.2575 (2013). 23892899

[b16] Seville L. A. *et al.* Distribution of Tetracycline and Erythromycin Resistance Genes Among Human Oral and Fecal Metagenomic DNA. Microb Drug Resist 15, 159–166, 10.1089/Mdr.2009.0916 (2009). 19728772

[b17] Ghosh T. S., Sen Gupta S., Nair G. B. & Mande S. S. In Silico Analysis of Antibiotic Resistance Genes in the Gut Microflora of Individuals from Diverse Geographies and Age-Groups. Plos One 8, ARTN e83823, 10.1371/journal.pone.0083823 (2013). PMC3877126 24391833

[b18] Moore A. M. *et al.* Pediatric Fecal Microbiota Harbor Diverse and Novel Antibiotic Resistance Genes. Plos One 8, ARTN e78822, 10.1371/journal.pone.0078822 (2013). PMC3827270 24236055

[b19] Wang H. *et al.* Antibiotic Body Burden of Chinese School Children: A Multisite Biomonitoring-based Study. Environmental Science & Technology 49, 5070–5079, 10.1021/es5059428 (2015). 25830781

[b20] Falagas M. E. & Kampoti M. Obesity and infection. Lancet Infect Dis 6, 438–446, 10.1016/S1473-3099(06)70523-0 (2006). 16790384

[b21] Gibbs H. *et al.* The impact of obesity on drug prescribing in primary care. Brit J Gen Pract 55, 743–749 (2005). 16212848 PMC1562331

[b22] Longo C. *et al.* The effect of obesity on antibiotic treatment failure: a historical cohort study. Pharmacoepidem Dr S 22, 970–976, 10.1002/Pds.3461 (2013). 23733599

[b23] Forslund K. *et al.* Country-specific antibiotic use practices impact the human gut resistome. Genome Res 23, 1163–1169, 10.1101/Gr.155465.113 (2013). 23568836 PMC3698509

[b24] Forslund K., Sunagawa S., Coelho L. P. & Bork P. Metagenomic insights into the human gut resistome and the forces that shape it. Bioessays 36, 316–329, 10.1002/Bies.201300143 (2014). 24474281

[b25] Sales C. M. & Lee P. K. Resource recovery from wastewater: application of meta-omics to phosphorus and carbon management. Current opinion in biotechnology 33, 260–267, 10.1016/j.copbio.2015.03.003 (2015). 25827118

[b26] Tadesse D. A. *et al.* Antimicrobial drug resistance in Escherichia coli from humans and food animals, United States, 1950–2002. Emerging infectious diseases 18, 741–749, 10.3201/eid1805.111153 (2012). 22515968 PMC3358085

[b27] Bouza E. & Cercenado E. Klebsiella and enterobacter: antibiotic resistance and treatment implications. Seminars in respiratory infections 17, 215–230, 10.1053/srin.2002.34693 (2002). 12226801

[b28] Tansarli G. S., Karageorgopoulos D. E., Kapaskelis A. & Falagas M. E. Impact of antimicrobial multidrug resistance on inpatient care cost: an evaluation of the evidence. Expert review of anti-infective therapy 11, 321–331, 10.1586/eri.13.4 (2013). 23458771

[b29] Qin J. *et al.* A metagenome-wide association study of gut microbiota in type 2 diabetes. Nature 490, 55–60, 10.1038/nature11450 (2012). 23023125

