# Supplementary material for: Diminution of the gut resistome after a gut microbiota-targeted dietary intervention in obese children
Source: Sci Rep. 2016 Apr 5;6:24030. doi: 10.1038/srep24030 (PMC4820771; doi:10.1038/srep24030)
Supplement: Supplementary Information [file srep24030-s1.pdf]

Diminution of the gut resistome after a gut microbiota-targeted dietary intervention in obese children

Guojun Wu, Chenhong Zhang, Jing Wang, Feng Zhang, Ruirui Wang, Jian Shen, Linghua Wang, Xiaoyan Pang, Xiaojun Zhang, Liping Zhao, Menghui Zhang *

Affiliation:

State Key Laboratory of Microbial Metabolism, Joint International Research Laboratory of Metabolic & Developmental Sciences, and School of Life Sciences and Biotechnology, Shanghai Jiao Tong University, Shanghai, 200240, P.R.China

* *Corresponding authors*: Menghui Zhang e-mail: mhzhang@sjtu.edu.cn

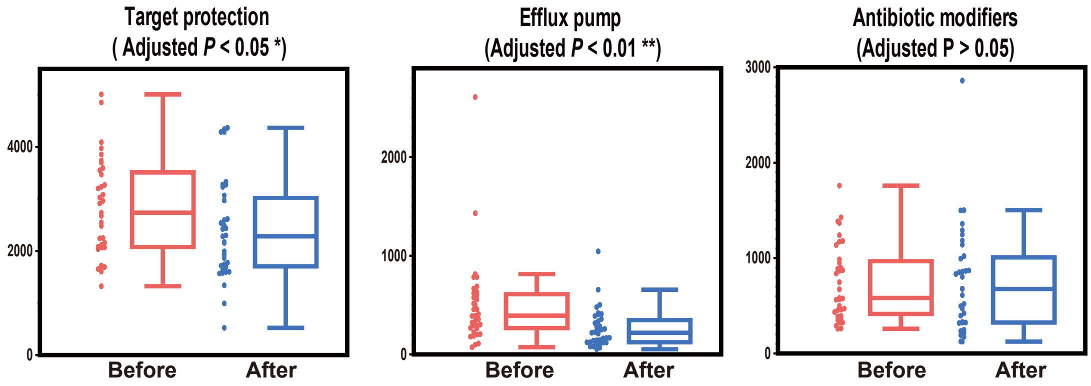

Supplementary Figure S1 | The altered abundances of ARGs acting via three different mechanisms. The ARGs were assigned to the three major resistance mechanisms according to ARDB. The abundances of ARGs conferring resistance for the same mechanism were summed. The boxes denote the interquartile range (IQR) between the first and third quartiles (25th and 75th percentiles, respectively), and the line inside the boxes denotes the median. The whiskers denote the lowest and highest values within 1.5 times of the IQR from the first and third quartiles, respectively. The samples are displayed as the dot on the left. Statistical analysis was performed with a two-tailed Wilcoxon matched-pairs signed rank test (n = 35). The P-values were adjusted (Benjamini & Hochberg 1995).

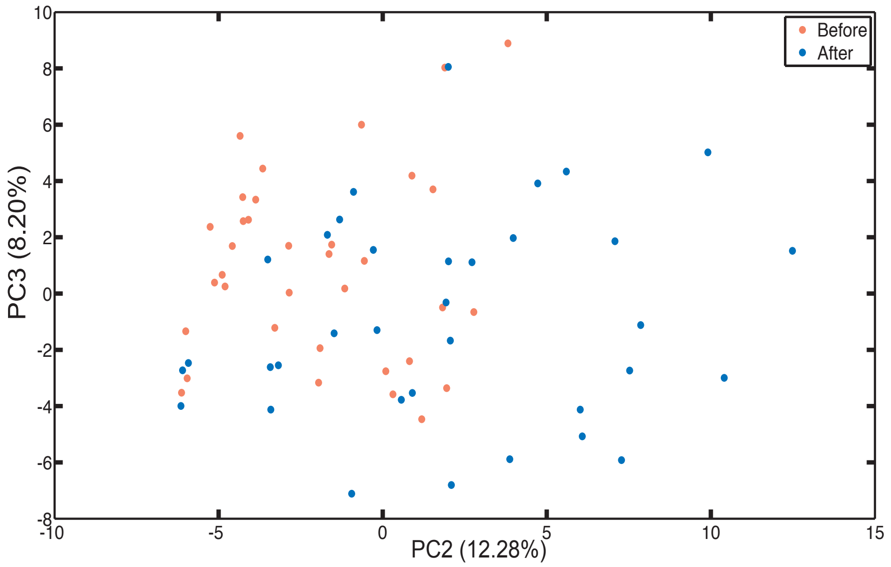

Supplementary Figure S2 | The altered profiles of ARG carriers after dietary intervention. PCA score plot based on the profile of 120 ARG carriers showing a trend for segregation between samples before and after dietary intervention (log-transformed, PERMONOVA P = 0.0584, permutation = 9999).

Gene ID	P-value[a]	Change	Gene type	Antibiotic	Antibiotic class	Mechanism
GD02_103489	0.010849027	decrease	bl1_ec	cephalosporin	beta_lactam	Antibiotic modifiers
GD02_105249	0.022748203	decrease	acrb	aminoglycoside,glycylcycline,macrolide,beta_lactam	aminoglycosides,glycylcycline,macrolide,beta_lactam	Efflux pump
GD02_110811	0.019754128	decrease	ermq	lincosamide,streptogramin_b,macrolide	lincosamides,streptogramin,macrolide	Target protection
GD02_112204	0.02754178	decrease	mdte	erythromycin	macrolide	Efflux pump
GD02_112205	0.011756398	decrease	mdtf	erythromycin	macrolide	Efflux pump
GD02_123177	0.01068977	decrease	macb	macrolide	macrolide	Efflux pump
GD02_127083	0.01068977	decrease	cata1	chloramphenicol	amphenicols	Antibiotic modifiers
GD02_158373	0.03738599	decrease	tet40	tetracycline	tetracyclines	Efflux pump
GD02_174171	0.046471785	decrease	teto	tetracycline	tetracyclines	Target protection
GD02_186961	0.006405585	decrease	tetc	tetracycline	tetracyclines	Efflux pump
GD02_23042	0.011117519	decrease	mdth	fosfomycin	other	Efflux pump
GD02_23054	0.017763681	decrease	mdtg	fosfomycin	other	Efflux pump
GD02_27563	0.010849027	decrease	mdfa	NA	NA	NA
GD02_39013	0.01068977	decrease	emrd	NA	NA	Efflux pump
GD02_41625	0.006405585	decrease	mdtk	enoxacin,norfloxacin	quinolone,quinolone	Efflux pump
GD02_45183	0.007269125	decrease	rosb	fosmidomycin	other	Efflux pump
GD02_45198	0.01663889	decrease	acra	aminoglycoside,glycylcycline,macrolide,beta_lactam	aminoglycosides,glycylcycline,macrolide,beta_lactam	Efflux pump
GD02_45199	0.008949203	decrease	acrb	aminoglycoside,glycylcycline,macrolide,beta_lactam	aminoglycosides,glycylcycline,macrolide,beta_lactam	Efflux pump
GD02_50698	0.01068977	decrease	arna	polymyxin	polypeptides	Target protection
GD02_57948	0.007269125	decrease	mdtm	chloramphenicol,norfloxacin	amphenicols,quinolone	Efflux pump
GD02_68989	0.01068977	decrease	tolc	aminoglycoside,glycylcycline,macrolide,beta_lactam	aminoglycosides,glycylcycline,macrolide,beta_lactam	Efflux pump
GD02_73580	0.01333727	decrease	baca	bacitracin	polypeptides	Efflux pump
GD02_82568	0.006405585	decrease	mdtl	chloramphenicol	amphenicols	Efflux pump
GD02_83077	0.01068977	decrease	bcr	NA	NA	Efflux pump
GD02_89355	0.022748203	decrease	mdtp	puromycin	aminonucleoside	Efflux pump
GD02_89356	0.01762169	decrease	mdto	puromycin	aminonucleoside	Efflux pump
GD02_89357	0.010849027	decrease	mdtn	puromycin	aminonucleoside	Efflux pump
GD02_97267	0.013767497	decrease	ksga	kasugamycin	aminoglycosides	Target protection
GD03_234422	0.027533673	decrease	aac6ie	isepamicin,netilmicin,tobramycin,amikacin,sisomicin,dibekacin	aminoglycosides,aminoglycosides,aminonucleoside,aminoglycosides,aminoglycosides	Antibiotic modifiers
GD03_269264	0.022840108	decrease	emrd	NA	NA	Efflux pump
GD03_274003	0.039971477	decrease	tetc	tetracycline	tetracyclines	Efflux pump
GD04_183056	0.047854853	decrease	vanrg	vancomycin	glycopeptide	Target protection
GD04_370449	0.01068977	decrease	acra	aminoglycoside,glycylcycline,macrolide,beta_lactam	aminoglycosides,glycylcycline,macrolide,beta_lactam	Efflux pump
GD06_23263	0.033551559	decrease	mdtm	chloramphenicol,norfloxacin	amphenicols,quinolone	Efflux pump
GD08_135131	0.029366258	decrease	bl2e_cepa	cephalosporin	beta_lactam	Antibiotic modifiers
GD08_152605	0.047854853	decrease	tetq	tetracycline	tetracyclines	Target protection
GD11_81761	0.006405585	decrease	ermg	lincosamide,streptogramin_b,macrolide	lincosamides,streptogramin,macrolide	Target protection
GD12_115363	0.01048363	decrease	acrb	aminoglycoside,glycylcycline,macrolide,beta_lactam	aminoglycosides,glycylcycline,macrolide,beta_lactam	Efflux pump
GD13_66504	0.046990477	decrease	mdth	fosfomycin	other	Efflux pump
GD13_88162	0.019898406	decrease	acra	aminoglycoside,glycylcycline,macrolide,beta_lactam	aminoglycosides,glycylcycline,macrolide,beta_lactam	Efflux pump
GD15_139317	0.007107035	decrease	tetc	tetracycline	tetracyclines	Efflux pump
GD15_245514	0.011117519	decrease	bl1_ec	cephalosporin	beta_lactam	Antibiotic modifiers
GD18_219448	0.047854853	decrease	tet32	tetracycline	tetracyclines	Target protection
GD20_105167	0.022817204	decrease	rosa	fosmidomycin	other	Efflux pump
GD20_121947	0.04029534	decrease	tetpb	tetracycline	tetracyclines	Target protection
GD20_71487	0.038642143	decrease	baca	bacitracin	polypeptides	Efflux pump
GD20_92301	0.047854853	decrease	ksga	kasugamycin	aminoglycosides	Target protection
GD21_110170	0.03691539	decrease	tetpb	tetracycline	tetracyclines	Target protection
GD23_145347	0.006405585	decrease	acra	aminoglycoside,glycylcycline,macrolide,beta_lactam	aminoglycosides,glycylcycline,macrolide,beta_lactam	Efflux pump
GD23_145348	0.007140725	decrease	acrb	aminoglycoside,glycylcycline,macrolide,beta_lactam	aminoglycosides,glycylcycline,macrolide,beta_lactam	Efflux pump
GD24_225038	0.006405585	decrease	tetc	tetracycline	tetracyclines	Efflux pump
GD28_103338	0.022840108	decrease	mdtg	fosfomycin	other	Efflux pump
GD28_138810	0.018240177	decrease	tolc	aminoglycoside,glycylcycline,macrolide,beta_lactam	aminoglycosides,glycylcycline,macrolide,beta_lactam	Efflux pump
GD28_144604	0.045070807	decrease	bcr	NA	NA	Efflux pump
GD28_61950	0.010849027	decrease	tetc	tetracycline	tetracyclines	Efflux pump
GD29_109842	0.047854853	decrease	teto	tetracycline	tetracyclines	Target protection
GD31_102048	0.010849027	decrease	baca	bacitracin	polypeptides	Efflux pump
GD31_147921	0.007269125	decrease	tetc	tetracycline	tetracyclines	Efflux pump
GD31_154298	0.037984003	decrease	tolc	aminoglycoside,glycylcycline,macrolide,beta_lactam	aminoglycosides,glycylcycline,macrolide,beta_lactam	Efflux pump
GD31_231624	0.010863375	decrease	mdfa	NA	NA	NA
GD31_256237	0.037984003	decrease	bl2a_okp	penicillin		Antibiotic modifiers
GD31_269207	0.03691539	decrease	ermb	lincosamide,streptogramin_b,macrolide	lincosamides,streptogramin,macrolide	Target protection
GD32_10102	0.020369035	decrease	acrb	aminoglycoside,glycylcycline,macrolide,beta_lactam	aminoglycosides,glycylcycline,macrolide,beta_lactam	Efflux pump
GD32_113960	0.049345271	decrease	mdth	fosfomycin	other	Efflux pump
GD32_140719	0.018097409	decrease	macb	macrolide	macrolide	Efflux pump
GD32_250806	0.022748203	decrease	vanug	vancomycin	glycopeptide	Target protection
GD32_26136	0.006405585	decrease	acra	aminoglycoside,glycylcycline,macrolide,beta_lactam	aminoglycosides,glycylcycline,macrolide,beta_lactam	Efflux pump
GD32_84470	0.02754178	decrease	mdtk	enoxacin,norfloxacin	quinolone,quinolone	Efflux pump
GD32_8579	0.024011769	decrease	arna	polymyxin	polypeptides	Target protection
GD36_144381	0.02852471	decrease	sul2	sulfonamide	sulfonamide	Target protection
GD36_225459	0.006405585	decrease	tetc	tetracycline	tetracyclines	Efflux pump
GD39_140471	0.012154458	decrease	mdtm	chloramphenicol,norfloxacin	amphenicols,quinolone	Efflux pump
GD40_197303	0.011117519	decrease	arna	polymyxin	polypeptides	Target protection
GD40_248676	0.008406279	decrease	acrb	aminoglycoside,glycylcycline,macrolide,beta_lactam	aminoglycosides,glycylcycline,macrolide,beta_lactam	Efflux pump
GD41_230207	0.010863375	decrease	acrb	aminoglycoside,glycylcycline,macrolide,beta_lactam	aminoglycosides,glycylcycline,macrolide,beta_lactam	Efflux pump
GD43_122542	0.006405585	decrease	mdtm	chloramphenicol,norfloxacin	amphenicols,quinolone	Efflux pump
GD43_91996	0.022184965	decrease	acrb	aminoglycoside,glycylcycline,macrolide,beta_lactam	aminoglycosides,glycylcycline,macrolide,beta_lactam	Efflux pump
GD50_155565	0.019754128	decrease	arna	polymyxin	polypeptides	Target protection
GD51_174300	0.00906287	decrease	tetc	tetracycline	tetracyclines	Efflux pump
GD52_146010	0.010849027	decrease	acra	aminoglycoside,glycylcycline,macrolide,beta_lactam	aminoglycosides,glycylcycline,macrolide,beta_lactam	Efflux pump
GD52_217520	0.006405585	decrease	acrb	aminoglycoside,glycylcycline,macrolide,beta_lactam	aminoglycosides,glycylcycline,macrolide,beta_lactam	Efflux pump
GD52_222708	0.006405585	decrease	mdtk	enoxacin,norfloxacin	quinolone,quinolone	Efflux pump
GD56_156703	0.01663889	decrease	acrb	aminoglycoside,glycylcycline,macrolide,beta_lactam	aminoglycosides,glycylcycline,macrolide,beta_lactam	Efflux pump
GD56_191144	0.022840108	decrease	acra	aminoglycoside,glycylcycline,macrolide,beta_lactam	aminoglycosides,glycylcycline,macrolide,beta_lactam	Efflux pump
GD58_215195	0.022840108	decrease	acrb	aminoglycoside,glycylcycline,macrolide,beta_lactam	aminoglycosides,glycylcycline,macrolide,beta_lactam	Efflux pump
GD59_221482	0.006405585	decrease	mdtm	chloramphenicol,norfloxacin	amphenicols,quinolone	Efflux pump
GD02_205799	0.003729865	increase	pbp1a	penicillin	beta_lactam	Target protection
GD04_112145	0.000213488	increase	pbp1a	penicillin	beta_lactam	Target protection
GD08_240415	0.003591822	increase	pbp1a	penicillin	beta_lactam	Target protection
GD17_122282	0.000214171	increase	ermx	lincosamide,streptogramin_b,macrolide	lincosamides,streptogramin,macrolide	Target protection
GD24_231729	0.009789412	increase	pbp1a	penicillin	beta_lactam	Target protection
GD32_160233	0.00396803	increase	tetl	tetracycline	tetracyclines	Efflux pump
GD35_157074	0.000154587	increase	ermx	lincosamide,streptogramin_b,macrolide	lincosamides,streptogramin,macrolide	Target protection
GD39_124849	0.000488281	increase	baca	bacitracin	polypeptides	Efflux pump
GD40_172807	0.005506385	increase	pbp2b	penicillin	beta_lactam	Target protection
GD51_86167	0.000776411	increase	baca	bacitracin	polypeptides	Efflux pump

a P value was calculated by two-tailed Wilcoxon matched-pairs signed rank test (n=35) and adjusted by Benjamini & Hochberg 1995

Supplementary Table S1 |The information of the 96 ARGs with significant changes.
